# Hierarchical synchrotron diffraction and imaging study of the calcium sulfate hemihydrate–gypsum transformation

**DOI:** 10.1107/S1600576723002881

**Published:** 2023-05-09

**Authors:** Michela La Bella, Rogier Besselink, Jonathan P. Wright, Alexander E. S. Van Driessche, Alejandro Fernandez-Martinez, Carlotta Giacobbe

**Affiliations:** a European Synchrotron Radiation Facility, 71 Avenue Des Martyrs, Grenoble 38040, France; b Univ. Grenoble Alpes, Univ. Savoie Mont Blanc, CNRS, IRD, IFSTTAR, ISTerre, Grenoble 38000, France; c Instituto Andaluz de Ciencias de la Tierra (IACT), CSIC-University of Granada, Armilla 18100, Spain; Australian Synchrotron, ANSTO, Australia

**Keywords:** scanning 3D X-ray diffraction, s3DXRD, phase contrast tomography, gypsum hemihydrate

## Abstract

The mechanism of hydration of calcium sulfate hemihydrate (CaSO_4_·0.5H_2_O) to form gypsum (CaSO_4_·2H_2_O) was studied by combining scanning 3D X-ray diffraction (s3DXRD) and phase contrast tomography (PCT) to determine *in situ* the spatial and crystallographic relationship between these two phases. From s3DXRD measurements, the crystallographic structure, orientation and position of the crystalline grains in the sample during the hydration reaction were obtained, while the PCT reconstructions allowed visualization of the 3D shapes of the crystals during the reaction. This multi-scale study unfolds structural and morphological evidence of the dissolution–precipitation process of the gypsum plaster system, providing insights into the reactivity of specific crystallographic facets of the hemihydrate. In this work, epitaxial growth of gypsum crystals on the hemihydrate grains was not observed.

## Abstract 2

A combined approach of scanning 3D X-ray diffraction and phase contrast tomography has been used to study the hydration of calcium sulfate hemihydrate to form gypsum. This approach was found to be a powerful tool that permitted – for the first time – coupling of detailed crystallographic and morphological information on the hemihydrate–gypsum transition observed *in situ*.

## 1. Introduction

Gypsum plaster, or stucco or ‘plaster of Paris’, is the material made by the hydration of calcium sulfate hemihydrate (CaSO_4_·0.5H_2_O) resulting in the formation of calcium sulfate dihydrate, or gypsum (CaSO_4_·2H_2_O). Gypsum plaster has been used as a construction material since the Neolithic period (de Brito & Flores-Colen, 2015 ▸), and it is an important material for construction owing to its low cost and the high availability of the raw materials. The global consumption of both crude and calcined gypsum from the first to the third quarter of 2022 has been estimated at around 44 500 000 metric tons (Mineral Industry Surveys, *Gypsum in the third quarter of 2022*). The cohesive properties attributed to the needle-like morphology of the crystals make gypsum plaster a useful material for structural applications, as well as internal coatings (Pedreño-Rojas *et al.*, 2019 ▸; de Brito & Flores-Colen, 2015 ▸), and as an additive for the setting of cement (Taylor Harry, 1997 ▸). The biocompatibility of gypsum makes it suitable for medical applications (*i.e.* dentistry and orthopaedics; Thomas & Puleo, 2009 ▸) and as an excipient in the pharmaceutical industry (Carretero & Pozo, 2009 ▸).

**Figure 1:**
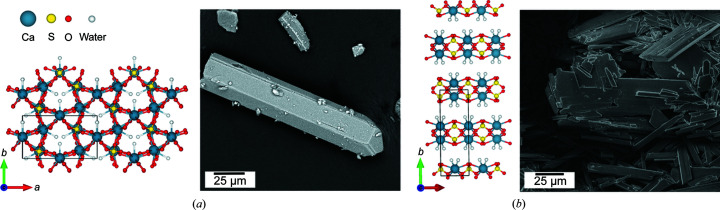
(*a*) Crystallographic structure of calcium sulfate hemihydrate (Ballirano *et al.*, 2001 ▸) along the *c* direction and SEM image of a typical α-hemihydrate crystal. (*b*) Crystallographic structure of calcium sulfate dihydrate (Boeyens & Ichharam, 2002 ▸) along the *c* direction and SEM image of an aggregate of gypsum crystals of different sizes and shapes. The SEM images have been taken with a VEGA3 TESCAN scanning electron microscope with a voltage of 16 kV. Legend: blue balls – Ca atoms; yellow balls – S atoms; red balls – O atoms; white balls – water molecules.

Calcium sulfate is present in nature as three different minerals: anhydrite (CaSO_4_), calcium sulfate hemihydrate or bassanite (CaSO_4_·0.5H_2_O), and gypsum (CaSO_4_·2H_2_O). The common structural characteristic for all these phases is the presence of chains of sulfate tetrahedra and calcium ions. In the case of gypsum, a layered structure is present with a hydrated interlayer that results in a preferential cleavage plane along the [010] direction. In the hemihydrate structure, the water molecules are located in water channels parallel to the [001] direction (Freyer & Voigt, 2003 ▸). Projections of the crystal structures of the hemihydrate [Fig. 1 ▸(*a*)] and gypsum [Fig. 1 ▸(*b*)] are given.

To obtain gypsum plaster, the hemihydrate is hydrated with aqueous solutions leading to the formation of gypsum plaster via the following exothermic reaction:

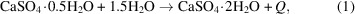

where *Q* is the heat generated by the exothermic reaction. As for most cementitious materials, and for gypsum plaster, the development of its strength is strongly related to its setting process (Lewry & Williamson, 1994 ▸). The dissolution of the hemihydrate continues to drive concentration gradients in the supersaturated solution while gypsum crystals are growing. The nucleation and growth rates of gypsum crystals have previously been followed by *in situ* X-ray tomography (Adrien *et al.*, 2016 ▸). The kinetics of these processes are controlled by thermodynamic drivers (saturation state of the interfacial water layers in the pores between the hemihydrate crystals) and kinetic barriers associated with the dissolution and precipitation pathways. Micro-textural properties also affect the hydro­dynamics and mixing of the aqueous solutions (Singh & Middendorf, 2007 ▸). Precise crystallographic studies of the hemihydrate–gypsum transformation, to determine which are the most reactive crystallographic facets during both hemihydrate dissolution and gypsum growth, should help us to control the plaster hydration reaction.

Hydration rates are known to alter the final porous microstructures of entangled gypsum crystals (Singh & Middendorf, 2007 ▸), but the models describing the crystallization of gypsum are still debated. Despite extensive study, it has been only in recent years, and due to non-destructive methods based on synchrotron X-ray radiation, that a non-classical nucleation model has been proposed to explain the early stages of gypsum crystallization (Van Driessche *et al.*, 2016 ▸). This complex process involves (i) the formation of a precursor phase, (ii) its aggregation and (iii) coalescence/reorganization forming gypsum crystals (Wang *et al.*, 2012 ▸; Van Driessche *et al.*, 2012 ▸; Saha *et al.*, 2012 ▸). A detailed examination of each of these steps (Stawski *et al.*, 2016 ▸) and the structural characteristics of the precursor units (Stawski *et al.*, 2019 ▸) can provide a complete view of the crystallization process.

Crystal nucleation and growth both determine the development of the microstructure of the final gypsum material. Due to the critical role that the evolution of the microstructure plays in the development of the properties of gypsum plaster, *in situ* observation of how the microstructure forms and sets is essential. Recent studies using X-ray tomography (with both laboratory equipment and synchrotron sources) have provided insights on the kinetics of dissolution and growth of the hemihydrate and gypsum crystals, and on the porosity of the material. The first *in situ* X-ray tomography study was carried out by Adrien *et al.* (2016 ▸) using laboratory tomography equipment and followed the kinetics of the reaction qualitatively and quantitatively. Recently, another *in situ* study probed the hydration reaction of α-hemihydrate using synchrotron X-ray tomography with unprecedented time (30–40 s) and space (0.163 µm voxel size) resolution (Seiller *et al.*, 2021 ▸). These studies provide a clear view of the morphologies of the grains involved in the reactions. However, crystallographic information is also needed to establish precise structure–reactivity–microstructure relationships.

In previous work (Artioli *et al.*, 2015 ▸, 2010 ▸; Claret *et al.*, 2018 ▸) focused on detecting calcium silicate hydrate (C-S-H), ettringite and carbonation during the hydration of cements, X-ray diffraction computed tomography was applied. In these cases, the diffraction spots were filtered out from the data to simplify the diffraction tomographic reconstruction problem and to focus on the role of the amorphous phases. In this paper, in order to follow the growth kinetics of larger crystals, it is instead essential to include these diffraction spots in the reconstructions. Scanning 3D X-ray diffraction (s3DXRD) is a recently developed synchrotron-based technique that can be used to locate and separate individual crystals in polycrystalline or grained (powdered) samples, and determine their size, shape and crystallographic orientation in three dimensions on the basis of X-ray diffraction patterns. The s3DXRD technique was originally developed for metallurgical research due to the high potential that multigrain crystallography offers for studying the evolution of polycrystalline materials (Hayashi *et al.*, 2015 ▸, 2014 ▸; Poulsen, 2004 ▸). The scanning method allows us to reconstruct structural features that are smaller than the size of the individual crystals. Differences between the surface and the bulk of the crystal can be resolved, as well as any other strain or orientation gradients. Grain or twin boundary locations and shapes within growing agglomerates can also be extracted from the s3DXRD reconstructions, whereas these are not detectable in tomography. The scanning method was previously applied on tin whisker growth (Hektor *et al.*, 2019 ▸; Henningsson *et al.*, 2020 ▸) with submicrometre spatial resolution where the initial and final states of phases and strains could be mapped in the same sample before and after annealing. Another recent application of s3DXRD is the work of Hayashi *et al.* (2019 ▸). They reconstructed intragranular stress tensors on bulk steel, providing a way to predict possible deformations of the material. The s3DXRD applications show the potential of s3DXRD to probe crystallographic and microstructural properties such as crystallite size and strain at the sub-micrometre scale, making it a perfect probe to determine the reactivity of polycrystalline and fine-grained materials.

Here, we used a combination of phase contrast tomography (PCT) and s3DXRD to follow the setting process of plaster *in situ*. PCT provides the morphological and spatial evolution of all the components of the hydration reaction. Crystallographic information such as phase, lattice and orientations of all the crystalline components of the system can be found by s3DXRD. The complementarity of these different methods gave multi-modal and multi-scale snapshots of the hydration reaction of gypsum plaster which provide detailed insights into the dissolution and precipitation reactions taking place.

## 2. Experimental

### 2.1. Materials and pre-characterization

A sample of α-hemihydrate was precipitated by mixing two equimolar solutions of Na_2_SO_4_ (Roth, >99%) and CaCl_2_ (Roth, >99%) in a 5 *M* solution of NaCl (Ossorio *et al.*, 2014 ▸). The solution was kept in an oven at 90°C for ∼1 h after mixing and was subsequently filtered with a vacuum-filtering system. The slurry obtained was washed several times with ethanol to minimize the presence of NaCl crystals. The sample was subsequently pre-characterized with scanning electron microscopy (SEM) and Fourier transform infrared spectroscopy (FTIR). SEM images provided qualitative observations about the shape and size of the hemihydrate crystals. A LEO 1530 (Gemini) scanning electron microscope at a voltage of 10 kV and 9 mm working distance was used. A small amount of sample was deposited onto a carbon tape mounted on an Al stub and gold-coated (50 nm). Images were then collected using the signal of both backscattered and secondary electrons. Mid-infrared FTIR spectra were collected to verify the purity of the samples. A Nicolet IS50R Research FTIR Spectrometer using the attenuated total reflectance sample holder with a single-reflection diamond crystal was used. Once the purity and the size of the crystals were verified, a quartz capillary with a 0.3 mm internal diameter was filled with the crystals. The top part of the capillary was filled with quartz wool fibres to keep the hemihydrate crystals in place during hydration. The hydration was then started by adding a saturated aqueous calcium sulfate solution to the capillary with a syringe in order to follow the hydration reaction of the sample *in situ*. The solution was added from the top of the capillary. The section of the capillary that was probed with X-rays was completely submerged in the solution, preventing the occurrence of transformation gradients.

### 2.2. Powder diffraction

**Figure 2:**
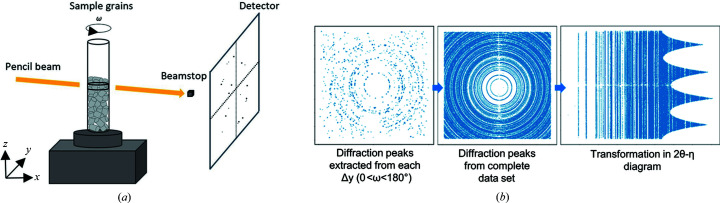
(*a*) Schematic representation of the experimental setup of the 3DXRD microscope at ID11 (ESRF, France). A glass capillary filled with calcium sulfate hemihydrate grains was mounted on the rotation stage and translated in the *y* direction. During the rotation, the diffraction spots coming from the grains were collected on the detector. A beamstop was placed in front of the centre of the detector in order to absorb the direct beam and prevent damaging the detector. (*b*) Segmentation method workflow with the *ImageD11* software: from the extraction of the diffraction peaks from one rotation (0 < ω < 180° at one Δ*y*), to the extraction of the peaks of the full data set, and finally the azimuthal integration of the diffraction pseudo-rings and representation of the peaks in the 2θ–η diagram.

In order to check the unit cell of α-hemihydrate for the s3DXRD indexing process, a high-resolution powder diffraction pattern was collected at ID22, the high-resolution powder diffraction beamline of the European Synchrotron Radiation Facility (ESRF), using a monochromatic X-ray beam at an incidence energy of *E* = 35 keV (λ = 0.35389 Å). Data were recorded at room temperature in the −5 to 30° 2θ range, using a combination of multi-analyser crystals and a 2D hybrid photon counting detector (Dectris Eiger2 X CdTe 2M-W), and merged together using the in-house software *id22sum* (Dejoie *et al.*, 2018 ▸). Le Bail fits implemented in *TOPAS* (version 5; Coelho, 2018 ▸) were then performed to confirm the unit cell of the hemihydrate. Powder diffraction patterns were also collected at ID11, the materials science beamline of the ESRF. Two data collections were performed, one of the starting dry hemihydrate and one after the hydration, to assess whether the hydration reaction had fully or partially taken place. The experimental setup was the same as that used for the s3DXRD (Fig. 2 ▸) with the exception that a beam size of 250 × 250 µm instead of 5 × 5 µm was used in order to give a better powder average. The patterns were collected in the 1–10° 2θ range at an energy of 43.575 keV (λ = 0.2843 Å). Preliminary instrumental calibration at ID11 was performed with a CeO_2_ powder standard using the *pyFAI-calib* tool and integration with *pyFAI-integrate* (Ashiotis *et al.*, 2015 ▸).

### 2.3. S3DXRD

*In situ* s3DXRD experiments were conducted at the 3DXRD station of ID11 (Poulsen, 2004 ▸). The analysed specimen contained calcium sulfate hemihydrate grains in a glass capillary. The first data collection was performed with the sample under dry conditions, and a second data set was taken 36 h after starting the hydration process. A monochromatic beam at an energy of 43.575 keV (Nd edge) was focused to a size of 5 × 5 µm using aluminium compound refractive lenses. A pencil beam approach was used, with a beam size significantly smaller than the average size of the grains. The sample was scanned horizontally along the *y* direction (across the beam direction) with a Δ*y* step equal to the beam width. In total 182 scans were acquired to probe one entire layer. Only one layer, 5 µm thick, was measured in the vertical direction (*z*). The two data sets were collected with an angular step of Δω = 1° over rotations between 0 and 180°, and an exposure time of 0.08 s. The acquisition of a complete data set took 2 h. The experimental approach was chosen as a compromise between the beam size and the exposure time. The parameters used permitted us to maintain a good spatial resolution and reduced the acquisition time. The detector used was a FreLoN2k camera, with 2048 × 2048 pixels of 47.2 × 47.2 µm, placed 154 mm from the sample. The capillary was mounted with its longitudinal direction parallel to the *z* direction as represented in the scheme in Fig. 2 ▸(*a*). The diffraction spots coming from the rotation of the grains were collected as 2D images from the detector. A schematic representation of the s3DXRD data acquisition is shown in Fig. 2 ▸(*a*).

The s3DXRD data analysis consists of indexing and refinement of the grains. Jupyter notebooks, based on the *ImageD11* software which is part of the *FABLE* complete suite for 3DXRD data treatment (Wright, 2005 ▸; Sørensen *et al.*, 2012 ▸; Hayashi *et al.*, 2015 ▸), were used to perform the indexing and refinement tasks. *ImageD11* permits us to segment the diffraction spots from the detector images and determine their centre-of-mass positions. The scattering vectors relative to the diffraction spots are calculated from the calibrated instrument geometry. A schematic representation of the segmentation sequence used for the two data sets is shown in Fig. 2 ▸(*b*). After location of the spots for all the images, these were transformed into a 2θ–η diagram to identify the crystallographic phases and check for possible texture. For the hemihydrate sample (initial state), the majority of the grains could be indexed using the known unit cell. For the hydrated sample, only the largest gypsum crystals were indexed. This selection was carried out using a higher threshold of intensity during the segmentation that also allowed us to avoid problems with spot overlaps. By segmenting and indexing only the diffraction spots coming from the larger crystals of gypsum, the smaller grains were discarded. For the indexing process of both data sets, only selected rings for the appropriate phases were used to generate trial orientation matrices [UBI matrices that are the inverse of the conventional (UB) matrix (Busing & Levy, 1967 ▸)]. The hemihydrate grains were indexed using the (101) and (103) rings of the *I*2 hemihydrate setting (Ballirano *et al.*, 2001 ▸). Gypsum grains were indexed using the (020) and (1 10) rings of the *C*2/*c* gypsum setting (Boeyens & Ichharam, 2002 ▸). These specific rings were chosen because of their low angle position and because they contained a high number of peaks that led to indexing of several grains. Grains were only retained if they indexed a certain number of peaks (1000 for hemihydrate grains and 500 for gypsum grains). Then the orientation matrices and the centre of mass of the indexed grains were refined and the position and shape of the crystals in space were reconstructed within a grain map (Hektor *et al.*, 2019 ▸). The iradon transformation of the sinograms made by the indexed peaks of each grain gave the position and shape of the grains in the grain map.

### 2.4. PCT

*In situ* PCT measurements were performed during the same experiment at ID11. The energy was not changed from the s3DXRD measurements but the setup presented a few differences. Here, no focusing optics were used and the projections were recorded with an imaging detector [FreLoN4M camera with region of interest (ROI) of 500 × 550 pixels] placed 300 mm downstream from the sample to enhance the phase contrast. The relatively large distance was chosen in order to give good phase contrast between the different components of the sample while retaining good spatial resolution. A 10× magnification lens was used, providing a voxel size of 1.56 µm. Since PCT and s3DXRD data collections were acquired sequentially by changing the beam width and detector, the measurements were performed on the same sample. A first measurement was performed before the hydration of the sample in order to characterize the dry hemihydrate grains. The following measurements were taken every 30 min after the injection of the solution in the capillary. Each measurement took 10 min to be completed. The last measurement was acquired after 36 h of hydration to match the final s3DXRD data.

The projections of the sample were collected over rotations in ω = 0 < ω < 360° with a total of 500 images per rotation. The data sets were reconstructed using ESRF in-house software. *Tomwer* and *Nabu* were used to apply the dark- and flat-field corrections and subsequently reconstruct transverse slices of the sample with the filtered back projection algorithm (Shepp & Logan, 1974 ▸). In order to segment grains from the surrounding solution and air bubbles, the Paganin phase retrieval algorithm (Paganin *et al.*, 2004 ▸) was applied during the reconstruction. The most suitable delta/beta value was δ/β = 30, giving the best compromise between sharpness and contrast to segment phases in the images. The reconstructions were segmented to locate grains of hemihydrate and gypsum and to follow the evolution of the microstructure of the plaster during hydration. A few selected grains of both hemihydrate and gypsum, which were also indexed in the s3DXRD data, were segmented manually (with the ROI painter tool of the *Dragonfly* software). In this case, to allow a clearer enhancement of the edges of the grains, no Paganin filter was applied. Preliminary visualization of the images was performed using both *ImageJ*/*Fiji* (Abramoff *et al.*, 2004 ▸) and *Dragonfly* [version 2021.1; Object Research System (ORS) Inc. Montreal, Canada]. The 3D rendering of the volumes of the full sample and the single grains together with the calculation of the volumes and surface area of dissolving hemihydrate and growing gypsum crystals were achieved with *Dragonfly*.

## 3. Results

### 3.1. Pre-characterization results

**Figure 3:**
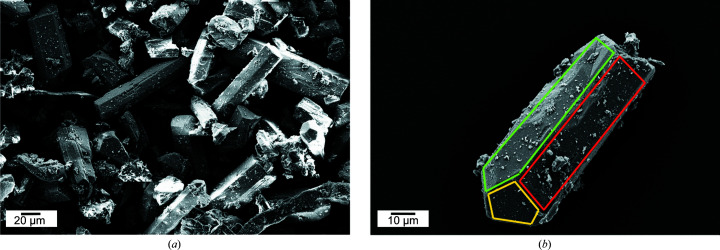
SEM images of the starting material synthesized and used in this work. (*a*) Agglomerate of α-hemihydrate crystals. (*b*) Single hemihydrate crystal, showing the typical crystal habit and pencil-shape tip of α-hemihydrate. Some crystallographic planes are recognizable: {111} facets in yellow, {100} facets in red, {011} facets in green.

SEM images of the hemihydrate crystals used as the starting material for the hydration show the typical shape and size associated with the α-hemihydrate phase (Singh & Middendorf, 2007 ▸). The crystal sizes shown in Fig. 3 ▸(*a*) are between 60 and 100 µm. Except for some small fragments, they all display an elongated and pencil-shaped crystal habit. In Fig. 3 ▸(*b*), one single grain of hemihydrate is shown. As expected for α-hemihydrate, it is characterized by a euhedral habit with well defined edges separating the different crystallographic faces. On the basis of the known α-hemihydrate crystal habit, it is possible to recognize three characteristic crystallographic planes: in yellow the {111} facets, in red the {100} facets and in green the {011} facets.

The starting material was also characterized using FTIR. The absorbance spectrum (shown in Fig. S1 of the supporting information) shows absorption bands in the regions of water scissoring (around 1596 cm^−1^) and OH stretching of water (3652 and 3756 cm^−1^) and in the region associated with the vibration of SO_4_^2−^ ions (around 1000–1200 cm^−1^). The spectrum is in agreement with previously reported calcium sulfate hemihydrate spectra (both α and β) and showed no contribution from gypsum (Bensted & Varma, 1971 ▸; Pons-Jiménez *et al.*, 2015 ▸).

### 3.2. PCT results

**Figure 4:**
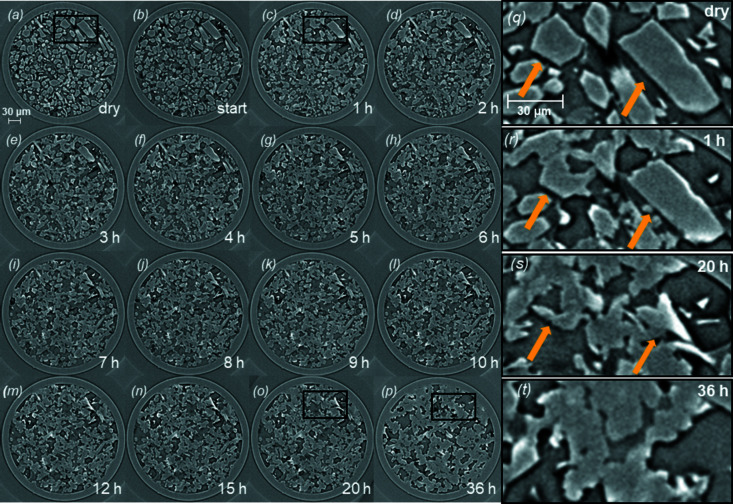
(*a*)–(*p*) Collection of slices at different hydration stages. The first slice corresponds to the sample in its dry starting condition. From the second slice to the last one 36 h of hydration have passed. As the hydration process takes place the initial hemihydrate grains progressively dissolve and small crystals of gypsum form and grow in an intricate network. (*q*)–(*t*) Zoom-in of a particular area of the sample, highlighted in (*a*), (*c*), (*o*) and (*p*), followed over the hydration reaction to show the dynamics of dissolution of two selected hemihydrate grains and the formation of gypsum crystals around them until the complete replacement of α-hemihydrate by gypsum.

From the reconstruction of the transverse slices of the capillary, it is possible to navigate inside the sample and select a region of interest to follow the evolution of a specific set of particles. In Figs. 4 ▸(*a*)–4 ▸(*p*) a collection of slices of the capillary is shown corresponding to the same layer of the sample scanned using s3DXRD. Each slice corresponds to a different time of hydration. From the first slice (dry hemihydrate) to the last (36 h of hydration), it is possible to observe the progressive dissolution of hemihydrate crystals and the formation of a network of smaller and interlocked gypsum crystals. The last slice [Fig. 4 ▸(*p*)] corresponds to the presence of mostly gypsum inside the capillary. Figs. 4 ▸(*q*)–4 ▸(*t*) show in detail the dissolution of two hemihydrate grains which were monitored until full dissolution. In Figs. 4 ▸(*q*) and 4 ▸(*r*) the two hemihydrate grains are clearly recognizable, in Fig. 4 ▸(*s*) they are almost completely dissolved, and in Fig. 4 ▸(*t*) the hemihydrate grains are replaced with a dense gypsum network. The choice of these two specific grains has been made because of their different orientations in the capillary. The first grain from the left is viewed parallel to its longitudinal axis, showing the pseudo-hexagonal shape of the crystal. The second particle displays the typical elongation of the α-hemihydrate crystals. In Fig. 4 ▸(*a*) the hemihydrate crystals show their original shape. In Fig. 4 ▸(*c*), after 1 h of hydration, the formation of small crystals of gypsum becomes visible around the larger hemihydrate grains. Moving to Fig. 4 ▸(*o*), corresponding to 20 h of hydration, the two hemihydrate grains are almost completely dissolved while the network of gypsum crystals further grows around the hemihydrate fragments. Fig. 4 ▸(*p*), corresponding to the last measurement after 36 h of hydration, shows the replacement of the hemihydrate by a complex matrix made of needles and plate-shaped gypsum crystals interlocked with each other.

**Figure 5:**
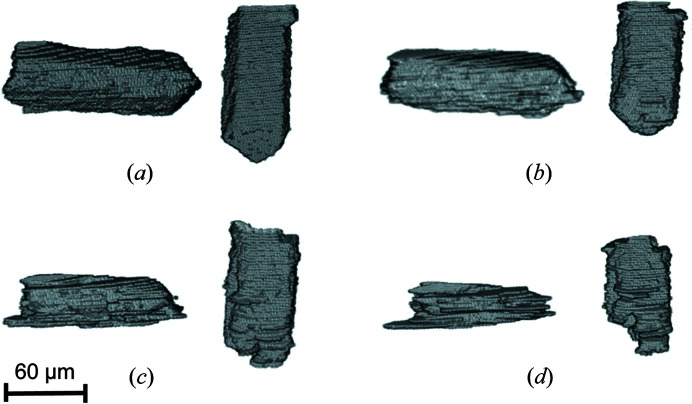
3D reconstruction of the two α-hemihydrate crystals with different orientations. The volume of the two grains was reconstructed in order to visualize the textural features of the dissolution process: (*a*) dry conditions, (*b*) 2 h of hydration, (*c*) 8 h of hydration, (*d*) 12 h of hydration.

In order to better visualize their dissolution in three dimensions, the two hemihydrate particles followed in Figs. 4 ▸(*q*)–4 ▸(*s*) were segmented and extracted from the whole volume. Fig. 5 ▸ shows the evolution of the 3D volumes of both hemihydrate grains throughout the hydration process, starting from the dry conditions [Fig. 5 ▸(*a*)], to 2 h of hydration [Fig. 5 ▸(*b*)], then to 8 h [(Fig. 5 ▸(*c*)] and finally to 12 h [Fig. 5 ▸(*d*)]. The reconstruction of the dissolution of the grains confirms what is already visible from the 2D slices in Figs. 4 ▸(*q*)–4 ▸(*t*). The two crystals are slowly dissolving, showing the formation of a layered texture at the surfaces that seems to suggest a preferential dissolution of the grains along the elongated axis. The two crystals maintain an elongated shape even when they are close to complete dissolution [Fig. 5 ▸(*d*)].

**Figure 6:**
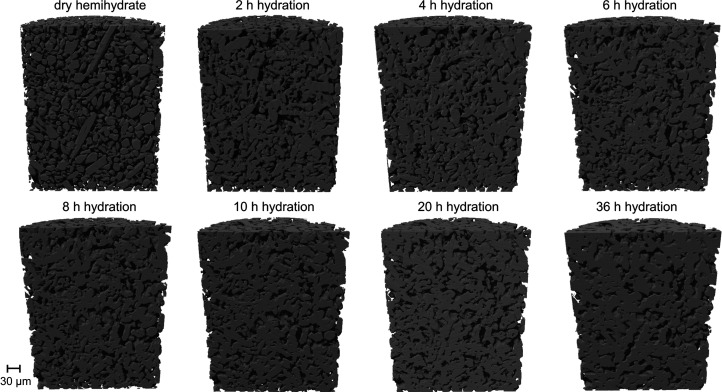
PCT 3D reconstructions of the volume of the sample during the hydration process. The volumes show the inside of the capillary during the development of the hydration process of the hemihydrate. The reconstructions start with a capillary filled with dry hemihydrate grains and end with the sample containing gypsum crystals.

After the reconstruction of the 3D volumes of each data set, they were segmented in order to discriminate between the different constituents of the sample. Fig. 6 ▸ shows a series of 3D reconstructions of half of the volume of the capillary over time. The volumes have been cut in half to show how the micro-texture evolves from the presence of only large hemihydrate crystals to the growth of gypsum. An evolution of the grain morphologies over time can be observed. The intermediate stages show how gypsum initially nucleates and grows in the pore spaces between the hemihydrate grains. The gypsum network starts to form with small agglomerates of needle-shaped crystals and ends with the presence of larger plate-shaped crystals.

### 3.3. S3DXRD results

#### 3.3.1. Grain maps

**Figure 7:**
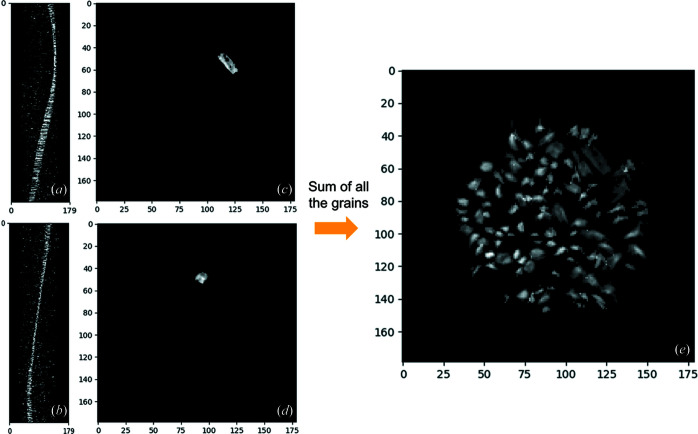
(*a*), (*b*) Sinograms of two hemihydrate grains. (*c*), (*d*) Reconstruction of the shape and position of the grains in the sample through iradon reconstruction of their sinograms. (*e*) Full grain map containing 107 indexed grains of dry hemihydrate. The greyscale represents the diffraction intensity of the grains.

Following the segmentation of the detector images of the dry hemihydrate data set, and the extraction of the diffraction spots, we indexed 107 hemihydrate grains using the monoclinic cell *I*2 refined by Ballirano *et al.* (2001 ▸). The indexing process works better for larger crystals because they produce stronger diffraction spots that are easier to locate, also providing more pixels for crystal shape reconstruction. The reconstruction of the shape and position of the grains in the grain map is achieved by iradon transformation of their sinograms. Since the sinograms of the grains are built from their indexed diffraction peaks, when a grain is large, the sinogram contains larger diffraction spots allowing a more accurate reconstruction of the shape. For this study, only the crystals that were large enough to offer meaningful shape information were added to the grain map for correlation with the PCT data. While building the sinogram of each grain, all the peaks that are close enough (depending on a chosen tolerance) to the *hkl* values of the rings selected are assigned to a specific grain via the indexing process. The indexing process associates pairs of peaks from both the rings that show geometrical relationships to the correspondent grain. The sinogram of a grain is built by the sum of the indexed peaks during rotation and shows their position at each angle between 0 and 180°. Once the iradon transformation was performed on the sinogram for each indexed grain, the reconstructions were treated to remove the background noise (*i.e.* peaks that are not coming from this grain). To do so, a threshold was applied to the intensity values of each pixel and all the pixels with a value lower than 0.02 were removed. In this way, it was possible to improve the reconstruction of the shape and position of each grain [Figs. 7 ▸(*c*)–7 ▸(*d*)]. Since several grains were indexed more than once, each reconstructed grain was checked and discarded in the case of doublets. The images in Figs. 7 ▸(*c*)–7 ▸(*d*) show the shape and position of the two grains already presented in Figs. 4 ▸(*q*) and 5 ▸(*a*). In the case of the first grain from the top [Fig. 7 ▸(*c*)], the longitudinal axis was lying parallel to the plane of the picture showing the elongation of the crystal. The grain in Fig. 7 ▸(*d*) was oriented perpendicular to the plane of the image, exposing a pseudo-hexagonal section of the crystal. When the reconstruction of all the 107 grains was completed, they were all summed in order to visualize the entire grain map of the hemihydrate [Fig. 7 ▸(*e*)].

From the indexing process of the gypsum data set, 102 grains were indexed using the *C*2/*c* lattice reported by Boeyens & Ichharam (2002 ▸). In this case, a higher intensity threshold, compared with the hemihydrate data set, was used for the segmentation process. This permitted us to index the largest gypsum grains. The grain map obtained from the indexing of gypsum grains is shown in Fig. S5.

#### 3.3.2. Orientation of the grains

The result of the indexing process is a matrix, UBI, that is the inverse of the conventional (UB) matrix (Busing & Levy, 1967 ▸). UBI links the *hkl* indices (**h**) of the crystallographic planes of the individual crystals with their scattering vectors (**g**) via

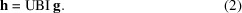

**Figure 8:**
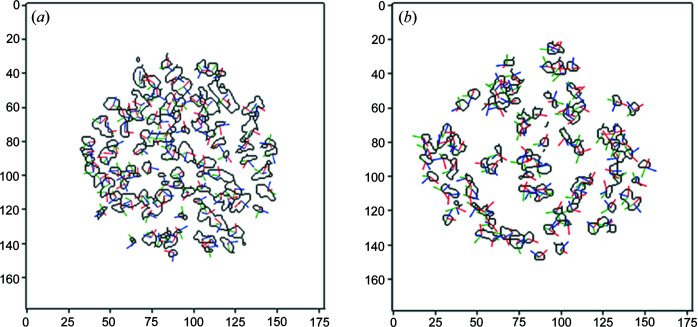
(*a*) Orientation of the three crystallographic axes of the hemihydrate grains, with the *a* axis represented in green, the *b* axis represented in blue and the *c* axis represented in red. (*b*) Orientation of the three crystallographic axes of gypsum grains, with the *a* axis represented in green, the *b* axis represented in blue and the *c* axis represented in red.

The crystal lattice vectors **a**, **b**, **c** in the sample coordinate system are given by the rows of the UBI matrix and plane normals can be derived via the dot product for any of the indexed hemihydrate and gypsum grains. The beam size used and the average size of the grains did not allow us to resolve possible orientation gradients within single grains. The main orientations of both hemihydrate and gypsum grains are shown in Fig. 8 ▸, where three vectors show the crystallographic directions of the unit cells of hemihydrate grains [Fig. 8 ▸(*a*)] and of gypsum [Fig. 8 ▸(*b*)].

## 4. Discussion

### 4.1. Gaining access to textural information at the mesoscale

**Figure 9:**
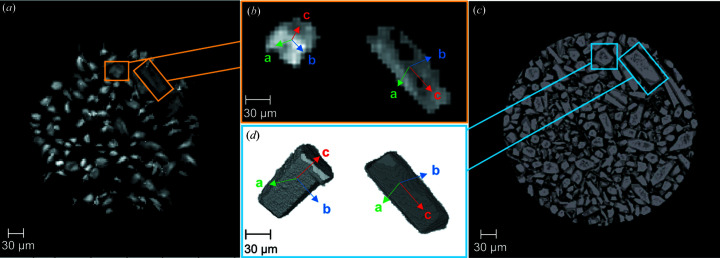
(*a*) S3DXRD grain map showing all the indexed hemihydrate grains. (*b*) Reconstruction of the two hemihydrate grains highlighted in (*a*) showing the orientation of the *a* (green), *b* (blue) and *c* (red) axes in space. (*c*) 3D reconstruction of a stack of three PCT slices that correspond to the same portion of the sample shown in (*a*). (*d*) 3D reconstruction of the two hemihydrate grains also shown in (*b*), obtained from the segmentation of the single grains, showing the orientation of the *a* (green), *b* (blue) and *c* (red) axes in space.

Previous work has highlighted the use of different X-ray tomography techniques to follow precipitation reactions in confined spaces (Godinho *et al.*, 2019 ▸, 2016 ▸; Anduix–Canto *et al.*, 2021 ▸). In comparison with these studies, the approach shown here presents a big advantage by introducing the crystallographic orientations of each indexed grain at the micrometre level. A direct correlation between the reconstructions made with s3DXRD (grain maps) and the reconstructions obtained from the PCT volumes can be established. For example, Figs. 9 ▸(*a*) and 9 ▸(*c*) show the indexed grains of dry α-hemihydrate reconstructed from s3DXRD and the grains obtained from PCT, respectively. All the grains shown in Fig. 9 ▸(*a*) match those present in Fig. 9 ▸(*c*). The majority of the smaller grains and fragments visible in the PCT reconstructions are not present in the s3DXRD grain map because the indexing process was tuned to select only the larger grains. Once the grains have been characterized using both techniques, it is possible to merge the most useful information from both, namely the orientation of the crystallographic planes, and the morphology and orientation in the space of the single grains. Fig. 9 ▸(*b*) shows two selected grains, reconstructed from the s3DXRD diffraction data, with different orientations. The result of the combination is shown in Fig. 9 ▸(*d*) where it is possible to identify in three dimensions the crystallographic orientations of the grains and to relate this to textural information from the 3D volume representation obtained from PCT. This allows us to confirm that the direction of the elongation of the hemihydrate grains is parallel to the [001] direction (Follner *et al.*, 2002 ▸).

### 4.2. Dissolution of α-hemihydrate

We can make use of our approach to gain structural and crystallographic insights of the changes observed in hemihydrate grains during the hydration process. If we look at Fig. 5 ▸ for example, it is possible to see how the morphology of the hemihydrate grains evolves during their dissolution, as they become thinner with time. Our combined approach allows us to establish the crystallographic directions of preferential dissolution at the scale of a single grain. The two grains shown in Fig. 9 ▸ are the same as those shown in Fig. 5 ▸. The observed microscopic ‘lamination’ of the grains occurs through the directions perpendicular to the [001] crystallographic direction. This observation matches with a recent study by Mishra *et al.* (2021 ▸), who introduced a force-field model of the CaSO_4_–H_2_O system and reported the cleavage and hydration properties of the different calcium sulfate phases. This study reported that the (010) and (100) planes in hemihydrate, both perpendicular to the [001] direction, are those with higher solid–water interfacial energies, and therefore are the less stable facets in water. An examination of the atomic structure of hemihydrate allows us also to determine that the number of bonds to be broken for ion removal from the (010) and (100) facets is the minimum compared with (001). This is in good agreement with the observed higher reactivity of those facets.

**Figure 10:**
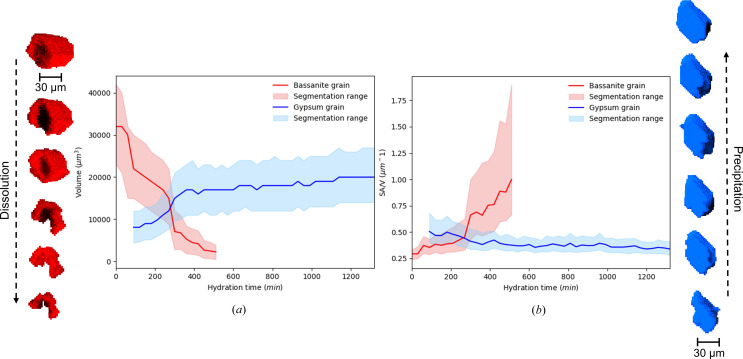
From the left: hemihydrate grain (in red) reconstructed from PCT segmentation showing the change in morphology due to dissolution. (*a*) Plot of the volume of the hemihydrate grain (red line) and gypsum grain (blue line). The range of segmentation is based on the values of the volume and surface area obtained by dilating and eroding the segmented grain. (*b*) Plot of the surface area, normalized for the volume, of the hemihydrate grain (red line) and the gypsum grain (blue line). The segmentation range is obtained in the same way as for (*a*). Gypsum grain (in blue) reconstructed from PCT segmentation showing the growth of the crystal during the hydration process of the sample.

The volumetric analysis of the PCT reconstructions of the dissolving hemihydrate grains provides a potential method of measuring the dissolution rate and can account for the evolution of the reactive surface area (Rufe & Hochella, 1999 ▸; Brandt & Bosbach, 2001 ▸) of bulk crystals, providing at the same time a 3D rendering of the changes in the morphology of the crystals. In Fig. 10 ▸ two plots are shown relative to the evolution of the volume [Fig. 10 ▸(*a*)] and normalized surface area [Fig. 10 ▸(*b*)] for one grain of α-hemihydrate and one grain of gypsum during the hydration process. The two particles were segmented manually, with the *Dragonfly* software, from the full volumes during the hydration in order to show some quantitative measurements coupled with the morphological evolution. The red particle on the left of Fig. 10 ▸ is the hemihydrate grain and the blue particle on the right is the gypsum grain which were used to calculate the volume and surface area variation during dissolution and growth. Since the two particles were segmented by hand, a segmentation range was considered. The upper limit of the segmentation range corresponded to the values of volume and surface area calculated by dilating the segmented particles. The lower limit of the segmentation range corresponded to the values calculated by eroding the segmented particles. It has been established that the rate of dissolution of crystals changes substantially according to the scale of the measurement. Rates of dissolution measured on bulk crystals of calcite, for example, were found to be higher than those measured on specific surfaces, probably because the crystal edges give a strong contribution to the dissolution owing to their dense concentration of defects (Dove & Platt, 1996 ▸; Arvidson *et al.*, 2003 ▸; Lasaga & Luttge, 2001 ▸). Macroscopic measurements therefore provide mean values and are insensitive to high degrees of heterogeneity in the dissolution rates (Peruffo *et al.*, 2013 ▸).

### 4.3. Precipitation of gypsum

**Figure 11:**
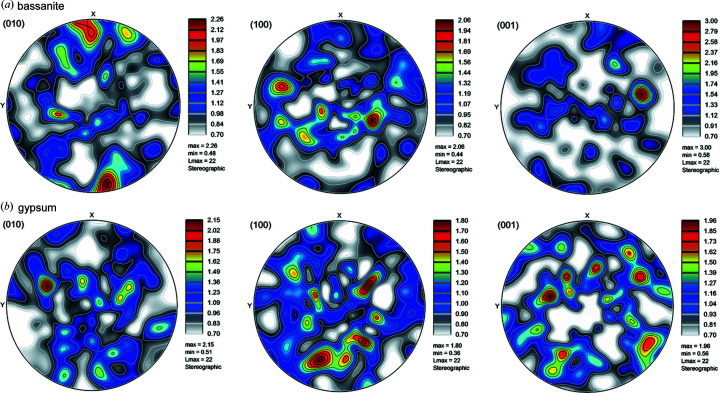
(*a*) Pole figures showing the distribution of the orientations of the starting hemihydrate grains for the directions [100], [010] and [001]. (*b*) Pole figures showing the distribution of the orientations of the final gypsum grains for the directions [100], [010] and [001]. The values associated with the colours of the pole figures indicate the density of the orientations. All the figures were produced using the software *ATEX* (Beausir & Fundenberger, 2017 ▸).

The hydration process involves a phase transformation that can occur via (i) a complete dissolution of the starting material and a precipitation of the final material, (ii) a coupled dissolution–precipitation driven by local supersaturation built up at the reacting interfaces, or (iii) epitaxial relationships between the initial and final materials that could happen via templating or surface precipitation (Noiriel & Daval, 2017 ▸). The information about the grain orientations obtained in this work can be used to ascertain the precise mechanism of transformation of hemihydrate to gypsum. To this end, pole figures showing the orientations of the hemihydrate grains at the start of the experiment and of gypsum grains at the end are shown in Fig. 11 ▸. The results indicate that both hemihydrate and gypsum crystals are randomly oriented and no specific relative orientation is found between the two. This indicates that epitaxially driven nucleation does not play a significant role in this system. Mechanisms (i) and (ii) are therefore possible. Given that the transport in the non-stirred capillary system used to contain the reactions is purely diffusive, it is likely that local gradients of concentration are present in the pore spaces between the grains, which could favour interfacial reactions such as those observed in other systems (Hellmann *et al.*, 2012 ▸, 2015 ▸).

**Figure 12:**
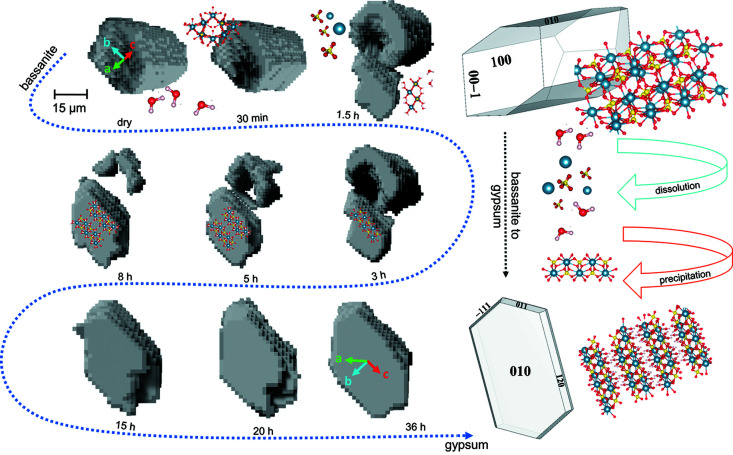
PCT reconstruction of segmented grains of the hemihydrate and gypsum during the dissolution–precipitation process. The reconstructions are reported following the time evolution (indicated by the blue dashed arrow), from the dry conditions (presence of hemihydrate only) to the end of the hydration (presence of gypsum only). The PCT reconstructions are coupled with the crystallographic orientations calculated for the two specific grains from the s3DXRD analysis. On the right is a schematic representation of the transformation from the hemihydrate structure to the gypsum structure. Legend: grey balls – Ca atoms; yellow balls – S atoms; red balls – O atoms; white balls – water molecules.

Since no crystallographic relationships that could demonstrate epitaxial growth have been found between the dissolving hemihydrate crystals and the growing gypsum crystals, we can state that the setting of gypsum plaster is a two-step process, as already seen by Adrien *et al.* (2016 ▸), involving the dissolution of hemihydrate and the precipitation of gypsum. An example of this process is shown in Fig. 12 ▸. The hemihydrate and gypsum particles shown in Fig. 12 ▸ were extracted from the portion of the sample that was probed with both PCT and s3DXRD. The two particles were spatially close to each other, most likely the result of the dissolution of hemihydrate and a local enrichment of the nearby pore solution that becomes supersaturated with respect to gypsum. Fig. 12 ▸ reports the dynamics of the dissolution–precipitation process relative to the two segmented particles of hemihydrate and gypsum. The reconstructions follow the evolution in time from the presence of dry hemihydrate to the formation of gypsum and the complete dissolution of hemihydrate. The hemihydrate grain starts to dissolve from the centre of the crystal, creating a pit that grows through the crystal along the [001] direction. The dissolution proceeds by dissolution of the pit walls {perpendicular to the [001] direction, highly reactive (100) and (010) planes}. In the last part of the dissolution the grain shrinks and adopts a ring shape. In our study, the nucleation of gypsum could not be observed due to the resolution limit, but growth of gypsum particles of >5 µm could be monitored. Depending on the formation conditions, calcium sulfate minerals can adopt a wide variety of crystal habits/morphologies and textures (Van Driessche *et al.*, 2019 ▸). The morphology of the crystal that is formed in this case is the plate, dominated by the {010} face.

## 5. Conclusions

The combination of the s3DXRD and PCT techniques was revealed to be a powerful tool to study reaction kinetics in heterogeneous materials, such as the hydration of α-hemihydrate to gypsum, allowing a multi-scale characterization of the system and providing a link between crystallographic and textural information. This approach allowed us to exclude epitaxial nucleation of gypsum crystals from the hemihydrate in our system, providing a 3D reconstruction of the dissolution–precipitation process *in situ*. The volumetric information from the PCT data and the orientation of the grains from the s3DXRD data enabled the identification of the reacting planes of the crystals. We have shown that the (100) and (010) planes of hemihydrate are preferentially dissolved, leading to the formation of a layered texture, most likely coming from the presence of structural defects in certain layers of the material. Indeed, structural defects (dislocations, point defects) are usually the loci where dissolution starts, given that the thermodynamic conditions are favourable (Saito, 1996 ▸; Lasaga, 1998 ▸; Luttge *et al.*, 2019 ▸; Lasaga & Luttge, 2001 ▸). Though a considerable amount of work has been carried out to monitor the dissolution of gypsum and anhydrite on specific surfaces on a local level (Mbogoro *et al.*, 2011 ▸; Peruffo *et al.*, 2013 ▸; Feng *et al.*, 2017 ▸; Bosbach & Rammensee, 1994 ▸), there are no local *in situ* investigations available on the dissolution of hemihydrate because of the speed of the process. In this work, we observed the dissolution of hemihydrate at the mesoscale, providing a link between microscopic (*e.g.* atomic force microscopy, atom-probe tomography, interferometry) and macroscopic studies of mineral weathering, thanks to the combination of information at the micrometre and single-grain scale. The results obtained in this study clearly show the suitability of this diffraction and tomography approach, paving the way for possible future investigations. The integration of the powder diffraction patterns from s3DXRD, for example, could reveal the presence of amorphous phases whereas a statistical volumetric analysis of the dissolving hemihydrate crystals could provide an estimation of the dissolution rate of the bulk sample.

## 6. Related literature

The following additional reference is cited in the supporting information: Abriel & Nesper (1993 ▸).

## Supplementary Material

Supporting figures. DOI: 10.1107/S1600576723002881/vb5051sup1.pdf
