# Supplementary material for: Hierarchical synchrotron diffraction and imaging study of the calcium sulfate hemihydrate–gypsum transformation
Source: J Appl Crystallogr. 2023 May 9;56(Pt 3):660–72. doi: 10.1107/S1600576723002881 (PMC10241062; doi:10.1107/S1600576723002881)
Supplement: Supplementary file 1 [file j-56-00660-sup1.pdf]

JOURNAL OF APPLIED CRYSTALLOGRAPHY

Volume 56 (2023)

Supporting information for article:

Hierarchical synchrotron diffraction and imaging study of the calcium sulfate hemihydrate-gypsum transformation

Michela La Bella, Rogier Besselink, Jonathan P. Wright, Alexander E. S. Van Driessche, Alejandro Fernandez-Martinez and Carlotta Giacobbe

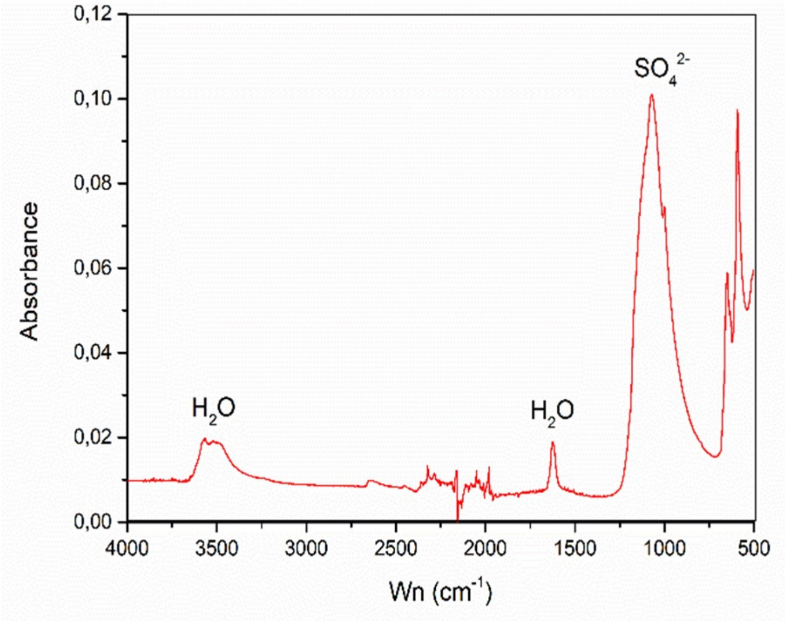

Figure S1 FTIR spectrum of the α-hemihydrate synthesized in this study.

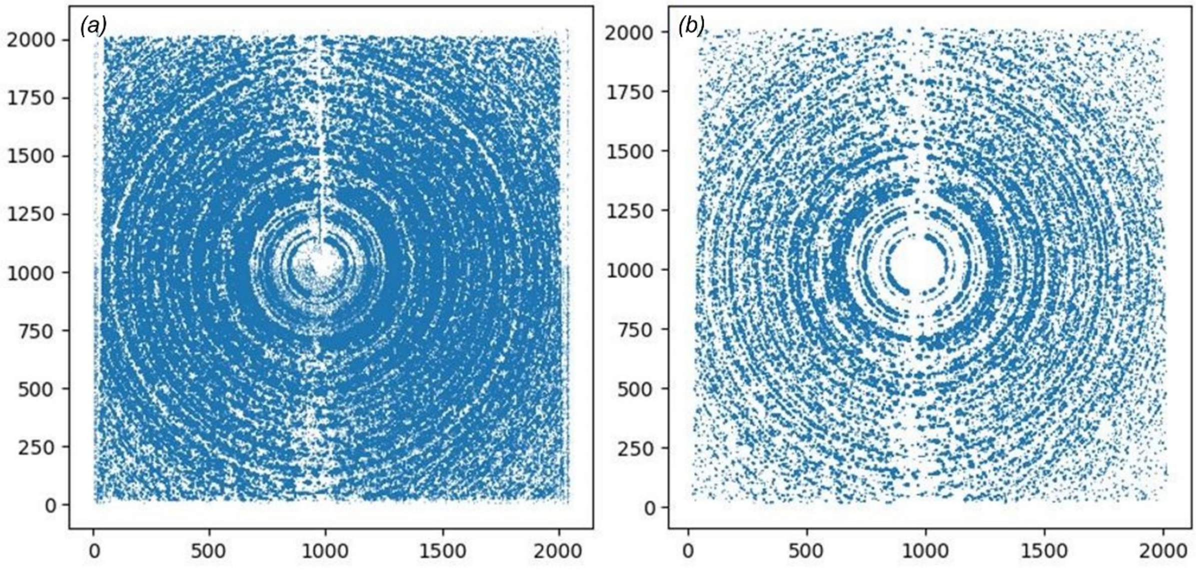

Figure S2 Peaks extracted from the segmentation process of *ImageD11* software: comparison between (a) low threshold value, (b) high threshold value.

Powder diffraction results

A Le Bail fit for the data collected at ID22 in the region 1.5-15° 2θ is shown in Figure S3a. Two different fits have been performed, one using the monoclinic space group I2 (Ballirano *et al.*, 2001) (Figure S3b) and another with the trigonal space group $P3_121$ described in Abriel & Nesper, 1993 (Abriel & Nesper,

1993) (Fig. S3c). The first one yielded the best fit and has been chosen as best model to describe our system. It is possible to see in the 2θ region 3.2-3.55° (Figure S3c) that the trigonal model fits the peak using only the reflection with Miller index {020}, while the monoclinic model is able to fit the three existing peaks (with Miller indexes {101}{-101}{200}). Figure S4 shows Le Bail refinements in the 2θ region 1.8-8° collected at ID11 prior to start the hydration and at the end of the process. These were carried out to confirm the purity of the starting materials (Figure S4a) and the completeness of the hydration process (Figure S4b). Figure S4a confirms that the hemihydrate used for this experiment is the monoclinic I2 α-hemihydrate. Figure S4b reveals that a small amount of hemihydrate was still present at the end of the hydration process. This amount is very small indicating that the reaction is largely complete. The presence of a small amount of hemihydrate at the end of the hydration process has been inferred from the presence of the small peak indicated with the letter B*. The experimental conditions used to acquire the powder patterns did not allow to perform a reliable Rietveld refinement so the percentage of hemihydrate still present has not been quantified.

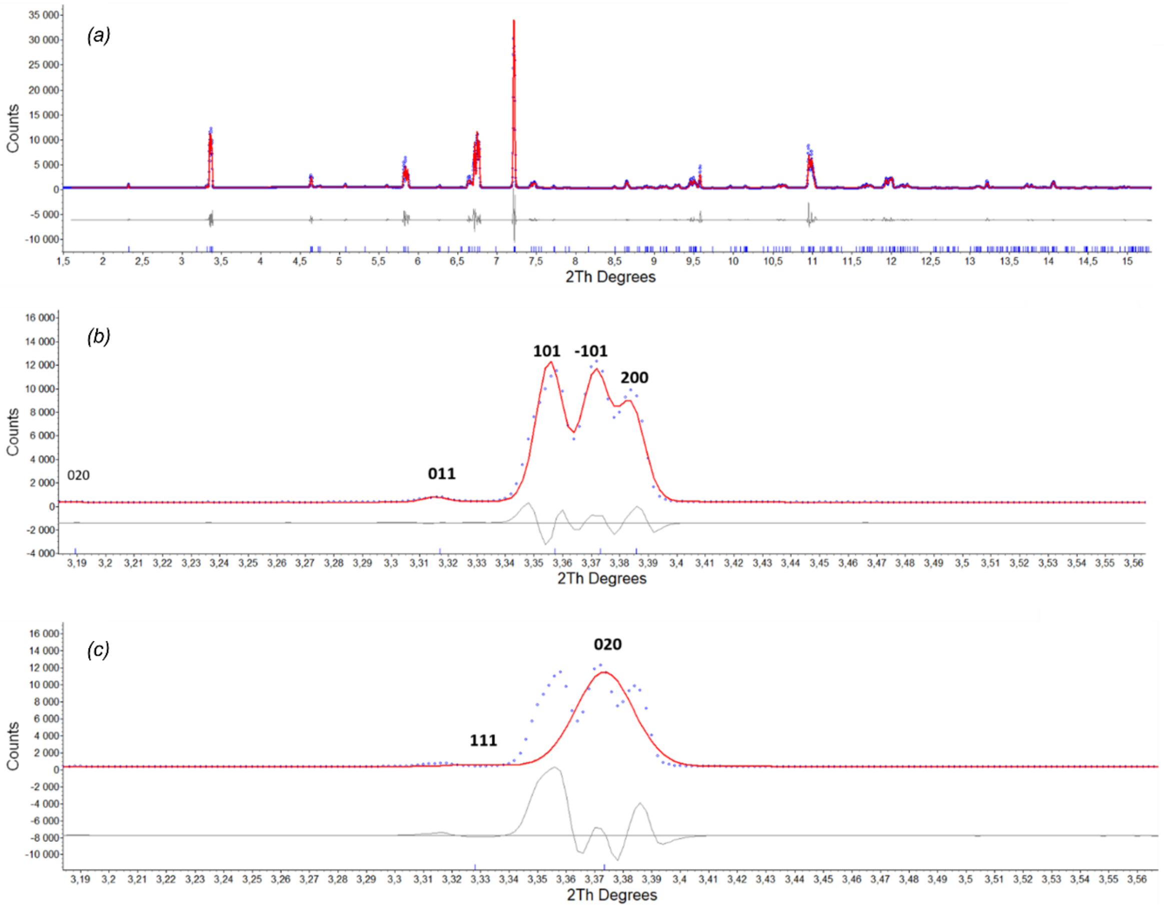

Figure S3 (a) Le Bail fit of the high resolution powder diffraction pattern collected at ID22 for the hemihydrate in the region 1.5-15° 2θ (experimental, blue dots, calculated, red continuous line). (b) Detail of the region 3.2-3.55 ° 2θ using the monoclinic I2 cell described in (Ballirano *et al.* 2001). (c) Detail of the region 3.2-3.55° 2θ using the trigonal $P3_121$ cell described in (Abriel & Nesper 1993).

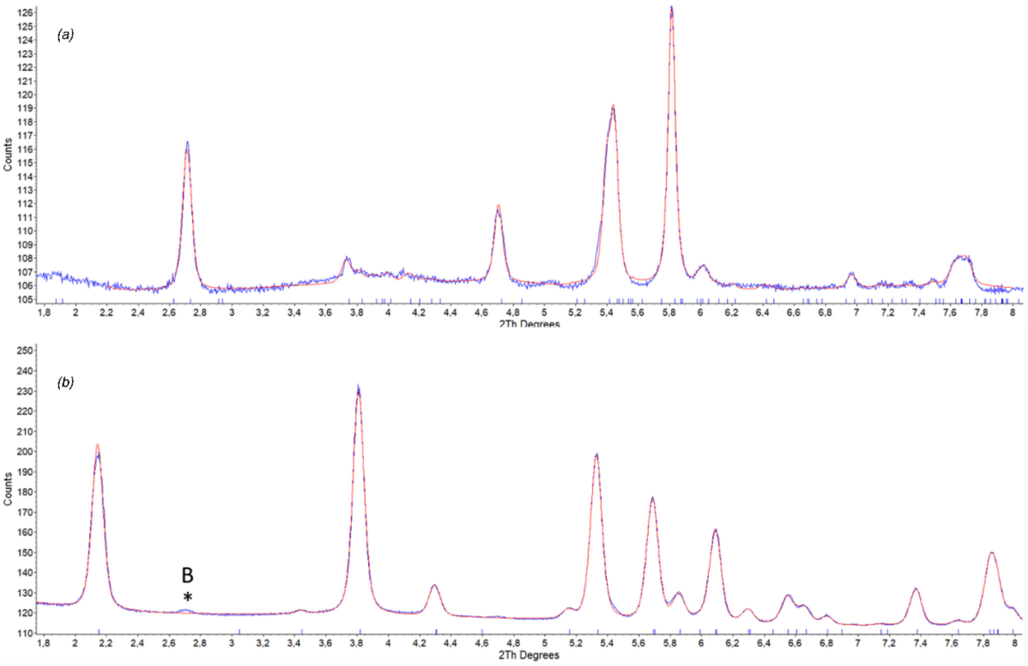

Figure S4 (a) Le Bail refinement of the powder diffraction pattern of the starting hemihydrate. The lattice parameters used are relative to the I2 structure (Ballirano *et al.* 2001). (b) Le Bail refinement of the gypsum (Boeyens & Ichharam 2002) obtained at the end of the hydration process. The remaining content of hemihydrate is negligible for the purpose of this study.

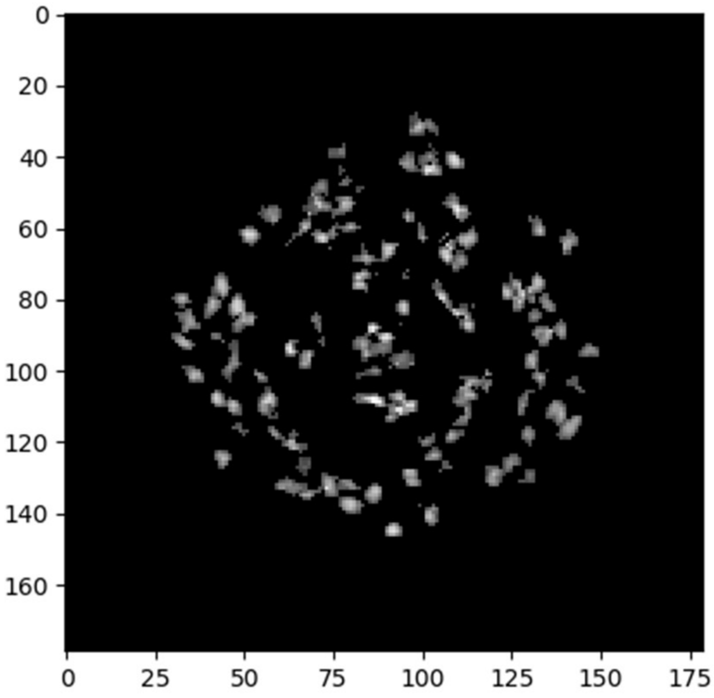

Figure S5 Grain map obtained from the indexing of 102 grains of gypsum.

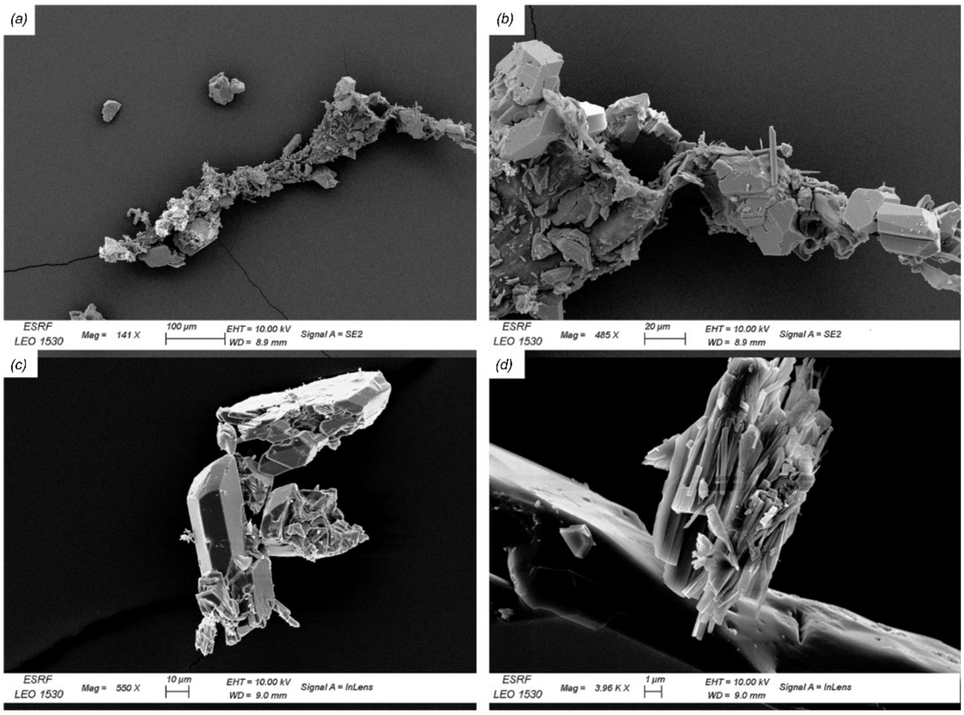

Figure S6 SEM images of the gypsum plaster formed after the hydration of hemihydrate that was monitored *in-situ*. (a) Portion of the gypsum crystals network extruded from the capillary. (b) Zoom on part of the sample showing different types of gypsum grains. (c) Detail of a tabular pseudo-rhombohedral gypsum crystal. (d) Detail of an agglomerate of gypsum needle shaped crystals.

References

Abriel, W. & Nesper, R. (1993). *Z. Krist.* **205**, 99–113.

Ballirano, P., Maras, A., Meloni, S. & Caminiti, R. (2001). *Eur. J. Mineral.* **13**, 985–993.

Boeyens, J. C. A. & Ichharam, V. V. H. (2002). *Z. Für Krist.-New Cryst. Struct.* **217**, 9–10.
